# The Submarine Volcano Eruption off El Hierro Island: Effects on the Scattering Migrant Biota and the Evolution of the Pelagic Communities

**DOI:** 10.1371/journal.pone.0102354

**Published:** 2014-07-21

**Authors:** Alejandro Ariza, Stein Kaartvedt, Anders Røstad, Juan Carlos Garijo, Javier Arístegui, Eugenio Fraile-Nuez, Santiago Hernández-León

**Affiliations:** 1 Instituto de Oceanografía y Cambio global, Universidad de Las Palmas de Gran Canaria, Las Palmas, Canary Islands, Spain; 2 Red Sea Research Center, King Abdullah University of Science and Technology, Thuwal, Makkah, Saudi Arabia; 3 Instituto Español de Oceanografía, Centro Oceanográfico de Canarias, Santa Cruz de Tenerife, Canary Islands, Spain; University of Connecticut, United States of America

## Abstract

The submarine volcano eruption off El Hierro Island (Canary Islands) on 10 October 2011 promoted dramatic perturbation of the water column leading to changes in the distribution of pelagic fauna. To study the response of the scattering biota, we combined acoustic data with hydrographic profiles and concurrent sea surface turbidity indexes from satellite imagery. We also monitored changes in the plankton and nekton communities through the eruptive and post-eruptive phases. Decrease of oxygen, acidification, rising temperature and deposition of chemicals in shallow waters resulted in a reduction of epipelagic stocks and a disruption of diel vertical migration (nocturnal ascent) of mesopelagic organisms. Furthermore, decreased light levels at depth caused by extinction in the volcanic plume resulted in a significant shallowing of the deep acoustic scattering layer. Once the eruption ceased, the distribution and abundances of the pelagic biota returned to baseline levels. There was no evidence of a volcano-induced bloom in the plankton community.

## Introduction

Submarine volcanoes are seabed fissures that spew mantle-derived materials and heat into the ocean. Erupted material mixes with the seawater and may thereby induce severe physical-chemical changes in the water column, in turn affecting the marine biota [1]–[4]. A submarine volcano eruption off El Hierro Island (Canary Islands) occurred from 10 October 2011 to 5 March 2012 at 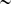
200 m depth 2 km south of La Restinga headland [5]. Fraile-Nuez et al. (2012) documented effects on the hydrography and the pelagic biota during the eruptive episode [6]. They reported divergent responses among picophytoplankton groups and the first observation of changes in the vertical distribution of the biota forming acoustic scattering layers. Santana-Casiano et al. (2013) described the chemical changes in the water from the onset of the volcano to the post-eruptive phase [7]. Their results showed strong deoxygenation and acidification across an extended region affected by the eruption, as well as increases in iron, nitrate, phosphate and silicate close to the volcano. Here, we focus on the effects of the volcano on the Deep and the Migrant Scattering Layers (DSL and MSL). We also report on changes in the plankton and nekton communities together with the volcano-induced abiotic perturbation. The study period spans from the most hostile scenario at the beginning of the eruption to the post-eruptive phase.

The DSL is acoustic backscatter from organisms inhabiting the mesopelagic zone [8], [9], the oceanic region between the base of the euphotic or epipelagic zone (100–200 m depth) and the top of the bathypelagic zone (1000 m depth) [10]. Part of this biota feeds during the night in the epipelagic zone, forming what we here refer to as the MSL. This so-called Diel Vertical Migration (DVM) occurs on a daily basis around the worlds oceans by large zooplankton and micronekton [11]–[13]. Abiotic factors including temperature [14], oxygen concentration [15], [16] and light irradiance [17], [18] play an important role in the distribution and behavior of these migrants, and they, in turn, have important implications for trophic connections and biogeochemical exchanges between the upper layers and the deep ocean [19]–[23].

Due to its offshore position far away from the NW African upwelling, El Hierro Island borders one of the most oligotrophic and transparent waters of the Canarian Archipelago [24], [25] and the epipelagic zone is characterized by low mesozooplankton densities and well-oxygenated waters [26]. Despite its low productivity, fisheries resources are abundant since the volcano-affected zone is a marine protected area, where only artisan fishing is allowed [27]. Furthermore, acoustic observations prior to the eruption [28] showed a dielly migrating DSL typically distributed between 400 and 700 m depth during daytime and forming strong scattering layers in the epipelagic zone at night.

The eruption promoted changes in the vertical structure of these acoustic scattering layers as a result of dramatically altered physical and chemical parameters. The sea surface acidified and warmed up, the surface oxygen was almost depleted and the waters became extremely turbid [6]. The perturbation affected the pelagic biota, and we here address changes in DVM patterns and the vertical distribution of the DSL. The eventual cessation of the volcanic activity allowed us to study the restoration of the plankton and nekton communities as well as the effects of the nutrient enrichment caused by the volcano emissions [7].

Here, we report on the relationship between acoustic scattering anomalies with concurrent sea surface turbidity data from satellite imagery and water perturbation as determined by oxygen profiles. We provide results from a six-month period, during and subsequent to the eruption episode, when levels of chlorophyll *a*, epipelagic mesozooplankton and vertical migrant micronekton were monitored.

## Methods

### Ethics statement

Field work was performed at the submarine volcano (27

37′07″N 017

59′28″W) and around the volcano-affected coast off El Hierro Island (Canary Islands) under a permit issued by the Spanish Government to the Spanish Oceanographic Institute. Studies did not involve endangered or protected species.

### Surveys

**Figure 1:**
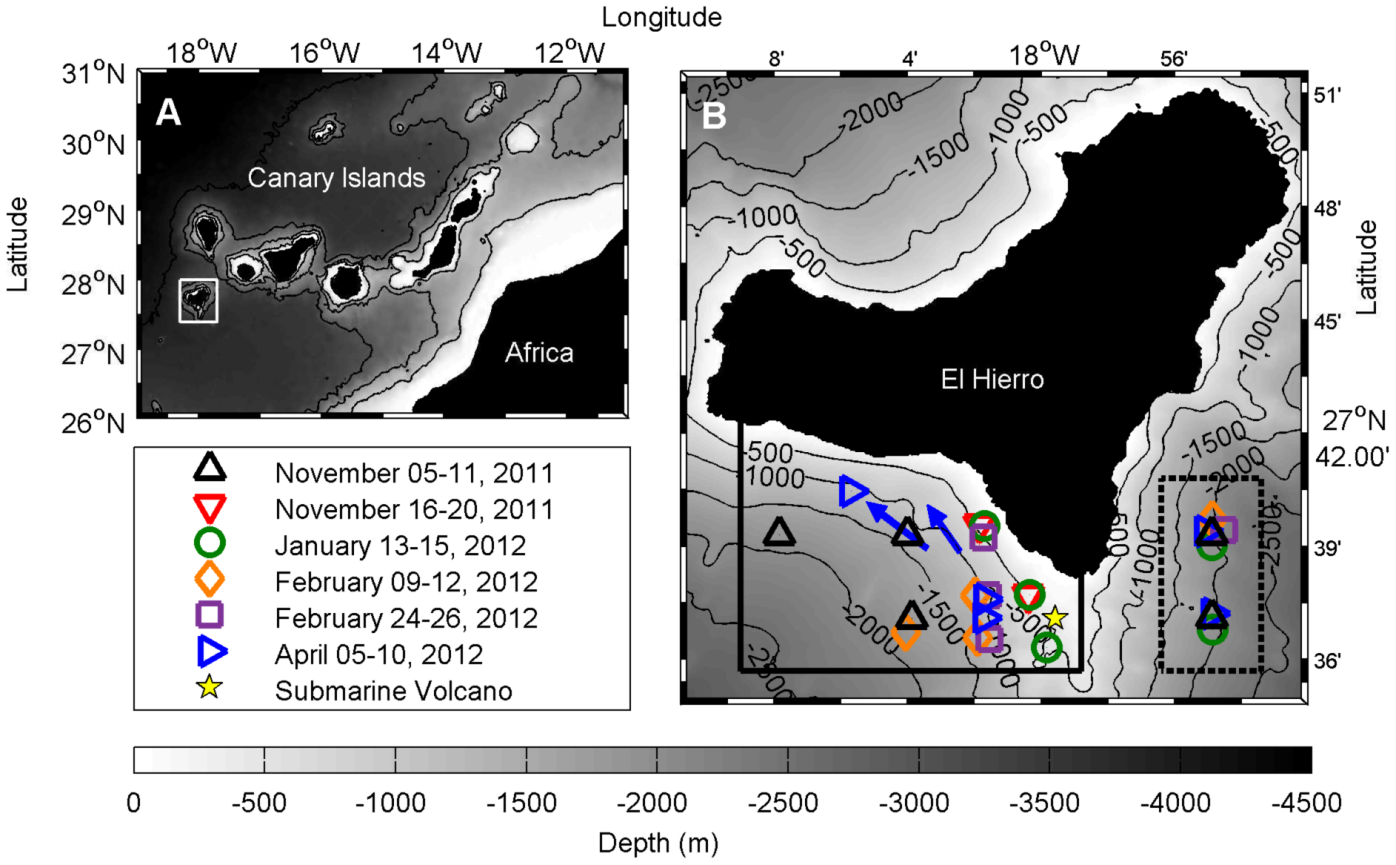
Position of El Hierro Island (A) and sampling points (B). The yellow star marks the position of the volcano. Symbols and arrows indicate oceanographic stations and locations of trawl tows, respectively. Colors refer to different surveys performed during the sampling period. Solid and dashed frames delimit experimental (volcano affected) and control (non-affected) zones, where satellite and acoustic data were collected along time-series.

Six cruises were conducted over the course of the eruptive and post-eruptive episodes (Fig. 1). On 5 November 2011, three weeks after the onset of the eruption, the Spanish Oceanographic Institute started operating the R/V *Ramón Margalef* southeast of El Hierro Island with the intention of monitoring the eruptive process. Five oceanographic surveys with hydrographic and biological samplings were performed until late February 2012 to study the effects of the volcano emissions on the pelagic biota. Epipelagic mesozooplankton and vertical migrant micronekton were assessed by plankton nets and acoustic sampling. Once the eruption stopped, a second oceanographic vessel, the R/V *Cornide de Saavedra*, performed the sixth and last survey in April 2012. This time, a pelagic trawl was included to collect larger organisms that had been detected by the echosounders throughout the previous cruises.

### Hydrography

Vertical profiles of conductivity and temperature were collected using a SeaBird 9/11-plus CTD equipped with dual conductivity and temperature sensors. The CTD sensors were calibrated at the SeaBird laboratory prior to the cruises. Water samples were obtained using a rosette of 24 10-liter Niskin bottles. In addition, a dissolved oxygen sensor (SeaBird SBE-43) and a fluorometer for chlorophyll *a* estimation (WetLabs ECO-FL) were linked to the CTD unit. Seawater analysis of dissolved oxygen (Winkler titrations) and Chlorophyll *a* extractions were performed to calibrate the voltage readings of both sensors. Analyses were carried out in accordance with the JGOFS recommendations [29]. Temperature and oxygen profiles of each survey were averaged from available stations within the volcano-affected area.

### Satellite Data

One kilometer spatial resolution data around El Hierro Island were used to monitor and visualize the evolution of the volcanic emissions. Data were obtained from the MODIS aqua sensor from the archive of the OceanColor Website (http://oceancolor.gsfc.nasa.gov). The standard product Sea Surface Reflectance (SSR, units: sr-1) at 667 nm was selected as a tracer of the volcanic plume since this spectral band has been found to be very effective for detecting turbidity anomalies in oceanic waters [30], [31]. Remote sensing of chlorophyll *a* was used as a proxy for phytoplankton concentrations, but only during the post-eruptive phase as this algorithm is based in the blue/green band ratio, which is also sensitive to non-living particles including suspended volcanic material [32], [33]. Due to cloudy weather, there was some missing data (empty pixels) from the satellite images. To overcome this lack of data, the daily images were averaged with both the prior and next day images. After that, remaining missing data were calculated by linear interpolation of the bordering pixels. Processing of satellite imagery was performed with SeaDAS software.

### Acoustic sampling

Hull-mounted SIMRAD EK60 echosounders (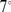 beam width) operating at 38 and 200 kHz were used for recording acoustic data (detection ranges of about 200 and 1000 m depth, respectively). The configuration was set at a 1024 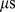 pulse duration and a 1 *s* ^−1^ ping rate. Acoustic data were not available for the upper 15 m due to the depth of the transducers (8 m on the R/V *Ramón Margalef* and 5 m on R/V *Cornide de Saavedra*) and because data down to 7 m below the transducers were excluded due to vessel-caused bubbles and near-field effects [34]. Since the volcano eruption was an unforeseen event, the R/V *Ramón Margalef* performed its first mission at sea without prior *in situ* acoustic calibration. For that reason, the acoustic data collected with this vessel were calibrated using correction factors obtained by standard calibration techniques [35] after the surveys. Echo sounders on the R/V *Cornide de Saavedra* were calibrated prior to the cruise, also using standard procedures.

### Net sampling

Epipelagic mesozooplankton was sampled from 200 m depth to the surface using a WP-2 plankton net equipped with 100 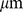 mesh. Samples were immediately fixed in 4% buffered formalin. In the laboratory, samples were digitalized using a scanner at a resolution of 1200 dpi and the organisms were automatically counted, measured and classified using ZooImage software according to the procedures described by Grosjean and Denis (2007) [36]. Taxonomic groups were established by a manually entered training set, achieving a global error rate of only 4.7% in the classification. The area of the organisms was then converted into biomass using the equations given by Lehette and Hernández-León (2009) [37].

Vertical migrant micronekton was captured using a midwater trawl with a 300 m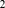 mouth area and 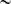
45 m length. The mesh size was 80 cm near the opening, decreasing to 1 cm in the cod end. In the last survey, two tows were performed obliquely in the MSL. The trawl was monitored by a Scanmar depth sensor and was guided into the MSL by information provided by the echosounders. The trawling speed varied between 2 and 3 knots and the effective fishing time was one hour. Samples were fixed in 4% buffered formalin for later identification and enumeration.

### Acoustic analysis

**Figure 2:**
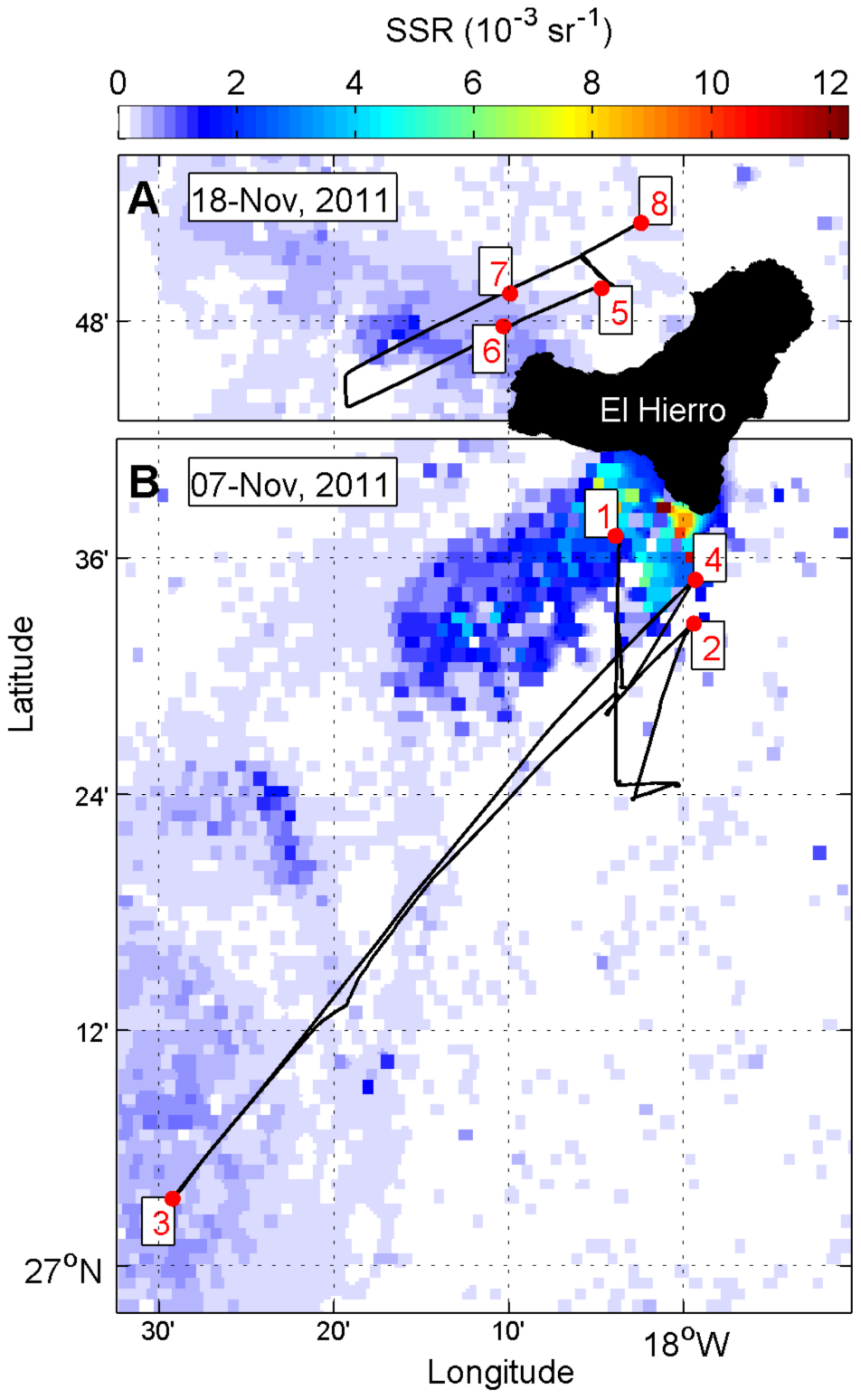
Sea Surface Reflectance (SSR) from satellite imagery. SSR indicates the degree of water turbidity and reveals the distribution of volcanic emissions. Red dots are CTD stations and black lines depict acoustic transects performed between them. Echograms corresponding to the transect lines and oxygen profiles from each station are shown in Fig. 3 (7 Nov.) and 4 (18 Nov.).

**Figure 3:**
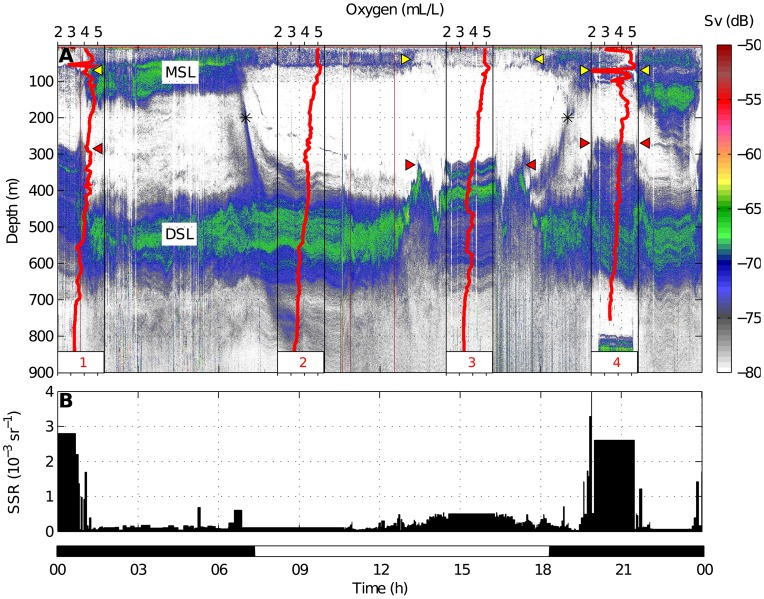
38 kHz echogram (A) and Sea Surface Reflectance (B) on 7 November 2011 along the acoustic transects indicated in Fig. 2b. The color scale refers to backscattering strength (Sv). Migrant and Deep Scattering Layers (MSL and DSL) as well as both diel migrations (*) are indicated. Acoustic anomalies are also indicated: the MSL weakening (yellow triangles) and the elevations of the DSL (red triangles). Red lines depict the dissolved oxygen profiles established during the acoustic track. The black color in the time scale refers to nighttime and white to daytime.

**Figure 4:**
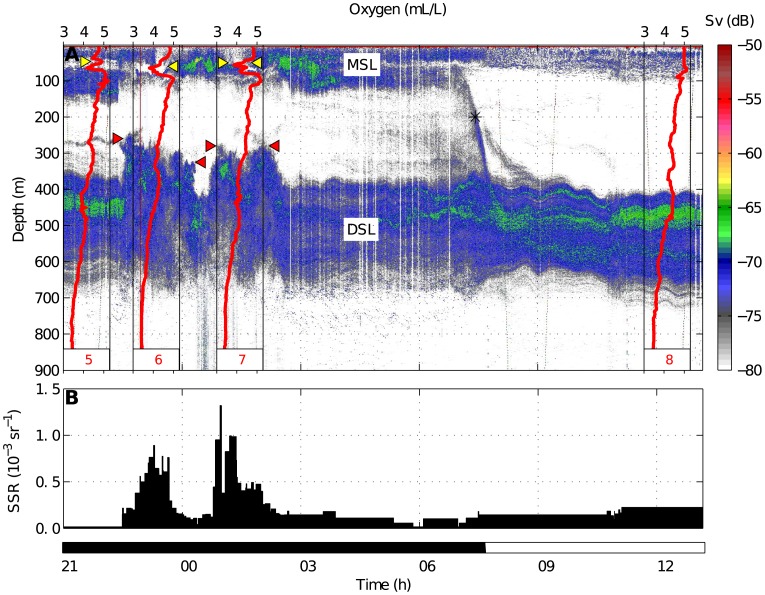
38 kHz echogram (A) and Sea Surface Reflectance (B) on 18 November 2011 along the acoustic transects indicated in Fig. 2a. The color scale refers to backscattering strength (Sv). Migrant and Deep Scattering Layers (MSL and DSL) as well as the downward diel migration (*) are indicated. Acoustic anomalies are also indicated: the MSL weakening (yellow triangles) and the elevations of the DSL (red triangles). Red lines depict the dissolved oxygen profiles established during the acoustic track. The black color in time scale refers to nighttime and white to daytime.

Anomalies in the distribution of acoustic targets were observed with the 38 kHz frequency related to the increment of surface turbidity and the oxygen depletion. For that reason, acoustic transects were made throughout the emission plumes, and their time-based echograms were displayed at this frequency together with corresponding latitude-longitude SSR from satellite imagery and spatially coincident profiles of dissolved oxygen. The acoustic measuring unit was the Volume Backscattering Strength (S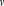, units: dB re 1 m^−1^) and the minimum detection threshold was set at −80 dB. Acoustic, satellite and hydrographic data were integrated and plotted using MATLAB software. In Fig. 2, two acoustic transects are shown: one between the main plume surrounding the volcano and an anticyclonic eddy, which was advecting emissions toward the open ocean (Fig. 3), and the other crossing a secondary plume, which drifted to the north side of the island (Fig. 4).

**Figure 5:**
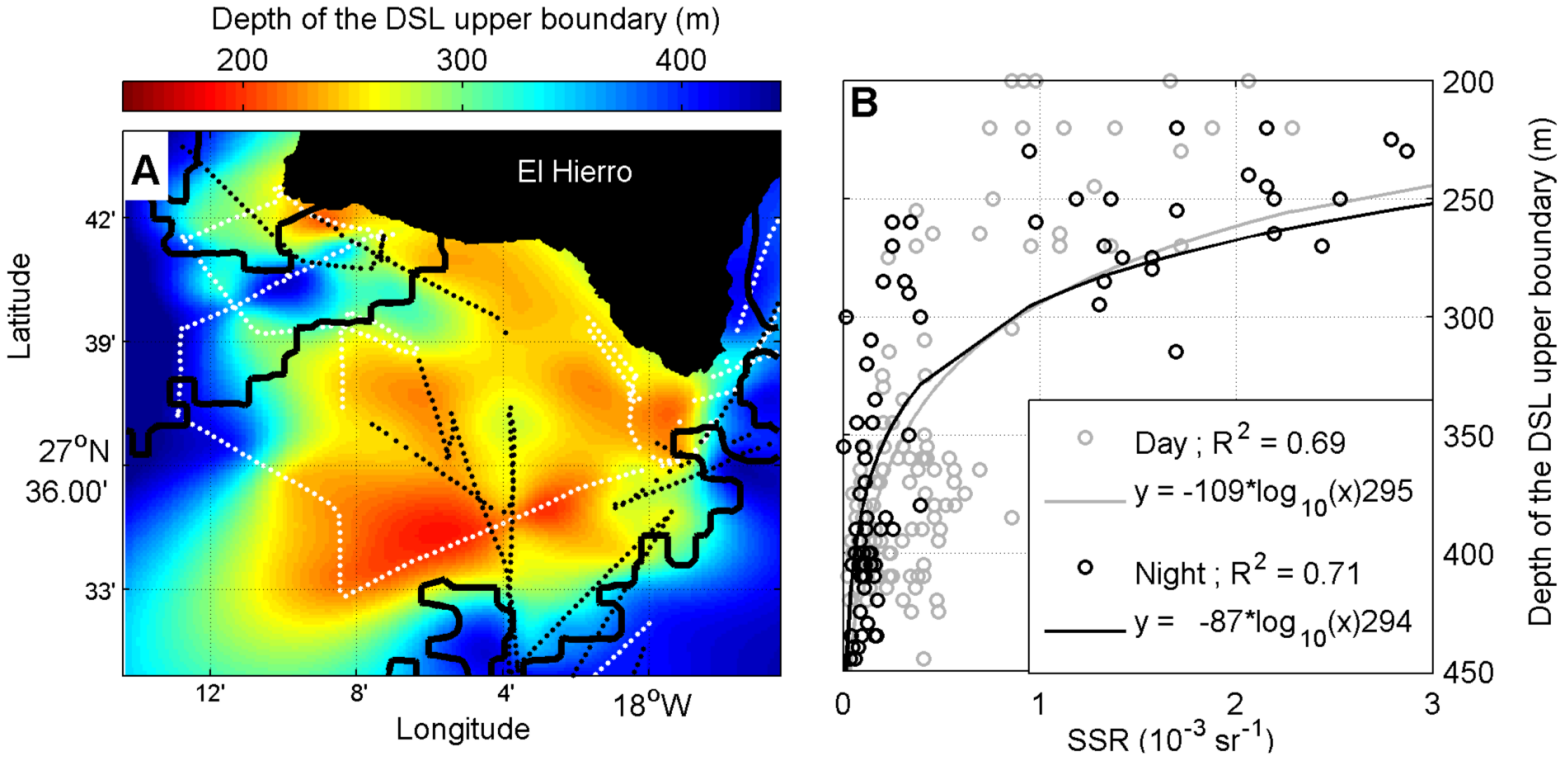
DSL depth throughout the volcanic plume. (A) Horizontal interpolation of the upper DSL depth. Dots indicate the positions of the original data points; the black ones refer to those collected during nighttime and the white ones during daytime. The solid black line depicts the outer contour of the volcanic plume. (B) Relationships between the upper DSL depth and the Sea Surface Reflectance (SSR, water turbidity proxy). Black and gray colors refer to data collected during night and daytime, respectively. Solid lines depict logarithmic regression curves fitted to data points. The vertical dashed line refers to the averaged SSR outside the volcanic plume.

The upper depth of the DSL (i.e., the part of the deep scattering apparently not involved with DVM) was mapped together with the contour of the plume to depict their geographical coupling. Since the upper part of the DSL appeared to be shallow beneath the plume (see Results), we manually traced the top boundary along the 38 kHz echogram using LSSS software. The averaged upper depth every 0.2 nm was afterwards map-projected and interpolated using the DIVA algorithm [38]. Day and nighttime DSL depths were interpolated together as we did not observe significant differences between the two. Nevertheless, original data points are shown in the map in black (night) and white (day) to distinguish them (Fig. 5a). The outer limit of the plume was set in the SSR isoline of 0.2 10^−3^ sr^−1^ because this was the averaged sea surface reflectance found in clean waters near the plume. In addition, the relationship between the DSL depth and the geographically coincident SSR was plotted and linear regressions were run for both day and nighttime values (Fig. 5b).

In addition to the net sampling (see above), the presence of epipelagic mesozooplankton and vertical migrant micronekton was also assessed using acoustics. The acoustic density of the former was calculated using the 200 kHz frequency, as the average length of members of this community (0.2–2 cm) fit quite well with its lowest resolution limit (wavelength of 0.75 cm). Fluid-like elongated mesozooplankton was the most abundant group collected by net sampling (see results). The detection threshold was therefore lowered to −100 dB in order to cover the weak backscattering caused by this group [39]. To exclude noise and echoes from mesopelagic migrants, only daytime acoustic data collected above 100 m depth were used and fish-like schools were removed before the echo integration. Vertical migrant micronekton (2–10 cm) was monitored by acoustic sampling, but during the last survey, the composition of this community was assessed by trawlings in the MSL. The MSL acoustic density was estimated with 38 kHz (wavelength of 3.9 cm) as that frequency has been shown to be optimal for fish detection [40], [41]. Fish was the main group forming the MSL in the Canary Islands according with our results and previous works [42], [43]. Only nighttime data collected above 200 m depth was used and the minimum threshold for integration was set at −80 dB to exclude weaker echoes caused by smaller organisms. Acoustic processing (noise removal and animal group allocation) was achieved using LSSS software [44], [45]. The averaged form of Sv, i.e., the mean volume backscattering strength (MVBS), was used as an indicator of animal density. We therefore refer to MVBS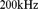 and MVBS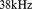 as proxies for the density of epipelagic mesozooplankton and vertical migrant micronekton, respectively. Because full species discrimination was not possible using the acoustics, these allocations should be interpreted as approximations based on the dominant communities found in net samples rather than exclusive taxonomic groups (i.e., macrozooplankton and gelatinous taxa were also expected to be within the MSL, but micronekton was the dominant group, and they were likely the most visible targets of the 38 kHz frequency).

### Monitoring of the pelagic biota

**Figure 6:**
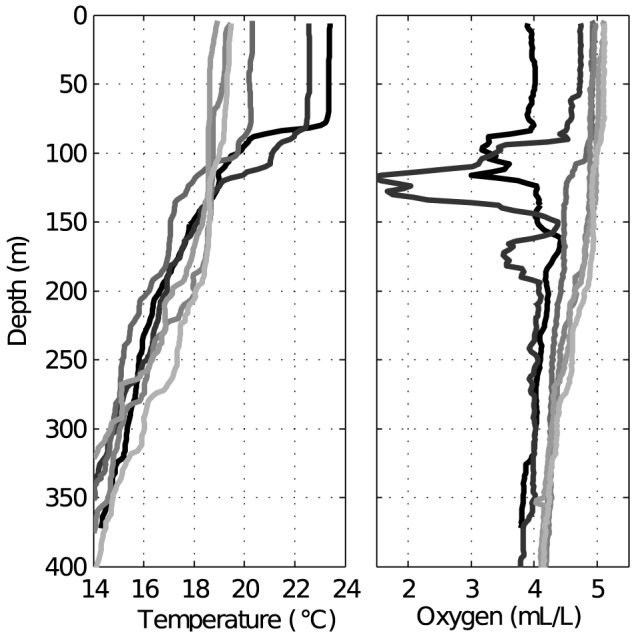
Temperature and dissolved oxygen profiles during the sampling period. The black lines indicate the first survey at the beginning of the eruption; the remaining surveys are indicated by progressively lighter gray lines (post-eruption) in chronologic order. Profiles were averaged from CTD casts performed within the affected zone.

**Figure 7:**
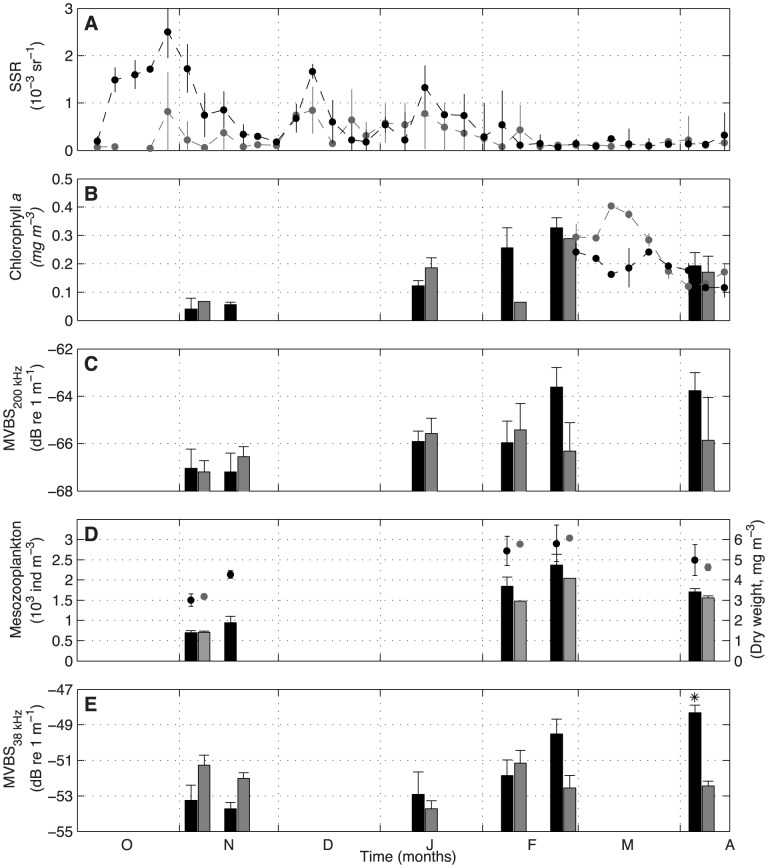
Time series with averaged parameters collected within the volcano-affected (black) and non-affected area (gray). (A) Evolution of the Sea Surface Reflectance (SSR) as an indicator of the degree of volcanic emissions over the sea surface. (B) *In situ* (bars) and remote sensing (dots) chlorophyll *a* concentration as a proxy of phytoplankton biomass. (C) Mean Volume Backscattering Strength at 200 kHz (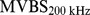) as a proxy of mesozooplankton biomass. (D) Mesozooplankton abundance (bars) and dry weight (dots) from WP-2 net hauls. (E) 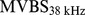 representing the acoustic density of the MSL (proxy of migrant micronekton biomass). Asterisk indicates that in the last survey, the composition of the MSL was characterized with concurrent trawling data (See Table 1).

Temperature, oxygen, chlorophyll *a*, epipelagic mesozooplankton and vertical migrant micronekton were measured during six months covering the eruptive and post-eruptive phases. As an indicator of the degree of volcanic emissions, five-day averaged SSRs are also shown (Fig. 6 and 7). Two zones were set for collecting samples and data: the experimental zone, in the south bay of the island where the erupted material remained blocked most of the time, and the control zone outside the bay, to the east of La Restinga front (see Fig. 1). Although the magmatic eruptive phase officially stopped on 5 March 2012 [5], waters along the south bay of the island were significantly cleaner around early February (according to SSR data). To study the effects on the surrounding pelagic biota, we designate the post-eruptive phase as starting in February 2012.

Chlorophyll *a* collected at 5 m depth and from remote sensing data was used as a proxy for the phytoplankton biomass. During the third and fifth cruises, *in situ* measurements of chlorophyll *a* were not available, so we derived the data from the fluometer sensor, which was calibrated using regression equations derived from chlorophyll *a* measurements performed during adjacent surveys. Epipelagic mesozooplankton and vertical migrant micronekton densities were assessed from both acoustic and net sampling approaches (see above). All those variables were replicated in the two zones. Data from water and net samplings were averaged from oceanographic stations that were selected within each zone. Satellite and acoustic data were also collected within those zones. The MVBS acoustic data were averaged from cells collected every 0.2 nm along the zones.

## Results

### Acoustic tracks and echograms

The 38 kHz echogram (Fig. 3) recorded during the acoustic transect on 7 November 2011 (Fig. 2b) was dominated by two scattering layers: the DSL at around 300–700 m depth during both day and night and the MSL above 200 m during nighttime. According to catches (Table 1) those scattering layers were composed of small fishes, cephalopods and shrimps, but they were largely dominated by myctophids. The echogram registered the upward and downward migrations of the MSL respectively coinciding with the sunset and sunrise. In addition to the typical DVM behavior, two anomalies were observed in the nocturnal echogram associated with the plume: a strong weakening of the MSL (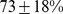) and an elevation of the upper limit of the DSL (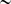
100–150 m). During the first and fourth CTD cast (in the plume) both anomalies occurred, coinciding with an increase of the SSR (the surface was more turbid) and a dramatic decrease in the dissolved oxygen around 70–80 m depth (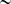
50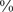). The second and third casts were performed during daytime, when we observed a weaker scattering layer above 50 m depth produced by non-migrant biota. This scattering layer and the DSL did not display anomalies at the second station (outside the plume), nor did the oxygen profile or the SSR. Nevertheless, at the third station (the anticyclonic eddy), the DSL increased again and the S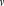 also decreased in shallow waters. That coincided with an increase of SSR but this time the oxygen profile remained unchanged.

**Table 1:** Averaged micronekton abundance within the MSL during the last survey.

Group	Family	Abundance (%)	
		Average	SD
Fishes	Myctophidae	71.30	2.28
	Gonostomatidae	1.33	1.22
Decapods	Oplophoridae	8.19	4.33
	Sergestidae	1.02	0.64
Cephalopods	Enoploteuthidae	11.16	9.99
Others	-	7.07	1.52

Data are shown in percentage of total abundance. Trawls are indicated as blue arrows in the map showed in Fig 1b.

According to remote sensing data, the plume was less dense on the northern side of the island by 18 November 2011 (Fig. 2a). The 38 kHz echogram (Fig. 4) revealed the same scattering layers as in previous results and the acoustic anomalies also occurred with an increase in SSR accompanied by hypoxia (see station 6 and 7). No anomalies were detected in the scattering biota where the waters remained clean and normoxic (station 8). In the case of the station 5, the oxygen sensor registered a decrease while SSR was quite low (no water dimming). Here, the MSL was depleted but the upper limit of the DSL remained in its normal depth range.

### DSL depth and SSR coupling

The upper limit of the DSL shown in Fig. 5a was markedly closer to the surface beneath the volcanic plume than in the surrounding non-affected waters. The upper fringe of the DSL was well above 200 m in the plume, but around 400 m outside. This pattern occurred both day and night and did not differ in magnitude with the diel cycle. Furthermore, the relationship between the depth of the DSL and the SSR followed a positive logarithmic curve, becoming shallower both during the day and night (Fig. 5b). It is noteworthy that a small increase in the sea surface reflectance, by about 0.3 10^−3^ units above the turbidity level under normal conditions, was enough to raise the DSL up to 300 m below the surface.

### Temporal changes in the pelagic biota

During the eruptive phase, the water column was characterized by a strong thermocline at around 80–90 m depth and high deoxygenation from 80 to 170 m depth (Fig. 6). The first sign of the eruption on the sea surface appeared on 12 October 2011, when the SSR increased from 0.1 10^−3^ to 0.5 10^−3^ sr^−1^, reaching the highest degree of water dimming by the end of October (0.8 10^−3^ sr^−1^). Around one week later, two consecutive biological samplings were performed (Fig. 7). Chlorophyll *a* was below 0.1 mg m^−3^ and mesozooplankton values ranged 700–950 ind m^−3^ and 3.0–4.2 mg m^−3^ (dry weight). The acoustic proxy for epipelagic mesozooplankton density (MVBS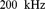) was below −67 dB while the vertical migrant micronekton (MVBS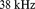) registered higher scattering levels, around −54 dB. Values for MVBS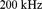 in the control zone (outside the plume) did not differ significantly from those collected in the plume, while MVBS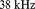 was lower inside the plume than in the non-affected area. According to SSR, mantle-derived materials continued spilling over the sea surface, but with progressively decreasing intensity pulses until the eruption stopped in early February. During this time, small turbidity pulses were also registered within the control zone.

Shortly before the end of the eruption, the concentration of *in situ* chlorophyll *a* in the volcano-affected area started to increase moderately but was still somewhat lower than in the control zone (January). Afterwards, chlorophyll *a* measurements were slightly higher in the affected area, reaching an average maximum of 0.33 mg m^−3^ during late February, when the eruption stopped. Remote sensing chlorophyll *a* measurements during March in the affected area were similar to *in situ* adjacent measurements, but slightly increased to 0.40 mg m^−3^ in the control zone. MVBS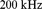 also started to recover by January, registering a relative maximum backscattering (−63 dB) coinciding with the chlorophyll *a* peak. The same pattern was found in epipelagic mesozooplankton abundances and biomass, where average maxima were 2370 ind m^−3^ and 5.8 mg m^−3^. MVBS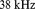 also increased, but the maximum average backscattering (−48 dB) was reached one month after the eruption ceased (early April). During the post-eruptive phase, both acoustic proxies revealed considerably higher backscattering in the volcano-affected zone compared with those in the control zone. It should be noted that relative peaks of all biological parameters also coincided with the breakdown of the thermocline and with normoxic conditions in the water column (See Fig. 6 from February on).

### Mesozooplankton and Micronekton composition

Concerning epipelagic mesozooplankton, no significant differences (Students t-test) were found when comparing the relative abundances of each taxa among the sampling surveys, nor between the experimental and control areas. Their averaged relative abundances are given for the whole sampling period (Table 2). Copepods largely dominated the community (94%), followed by chaetognaths (3%) and other organisms with abundances well below 1%.

**Table 2:** Averaged mesozooplankton abundance along the sampling period.

Group	Abundance (%)	
	Average	SD
Chaetognatha	3.01	1.22
Copepoda	94.23	1.50
Euphausiids like	0.29	0.22
Gelatinous	0.65	0.26
Others	1.98	0.69

Data are shown in percentage of total abundance.

The micronektonic composition (Table 1) corresponding to the MSL during the last survey was dominated by Lanternfishes in the myctophidae family (70%). The enoploteuthidae family were dominated by squids (11%), while shrimps from the oplophoridae family were the most abundant group of decapods (8%). Other minor groups also appeared but in quite low abundances (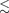
%).

## Discussion

### The weakening of the Migrant Scattering Layer

Based on the measured parameters, we related the weakening of the MSL to low oxygen concentrations in the upper layers. However, we consider the shallow hypoxia as a tracer of perturbations from the eruption rather than as a unique factor affecting the pelagic biota. Hypoxia was not the only perturbation observed in shallow waters during the submarine eruption. Fraile-Nuez et al. (2012) observed temperature anomalies in the vicinity of the volcano (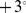
C at 75 m depth) and chemical compounds containing Fe, Cu, Cd, Pb and Al were observed at the sea surface [6]. Santana-Casiano et al. (2013) also reported that emissions of reduced sulfur compounds promoted a decrease in both the redox potential and the concentration of dissolved oxygen. Moreover, changes in the carbonate system contributed to the water acidification (−0.5 units at 75 m depth along the plume) [7].

The depletion of the scattering biota was evidence of the harmful effects of the volcano. By comparing the acoustic densities of the volcano-affected MSL with the neighboring non-affected zones, we observed that the former had a MVBS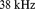 that was 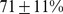 lower than that of the latter. That was partially balanced in the mesopelagic zone since the MVBS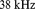 of the DSL was 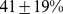 higher in the affected areas than in the non-affected areas. It seems feasible that part of the normally migrant biota remained in the midwaters when the emissions covered the surface. Nevertheless, another part (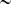
30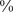) just disappeared from the ensonified volume. This disappearance could be explained by (1) horizontal migrations to find surrounding clean waters, as well as by (2) mortality caused by extreme physical-chemical perturbations. Support for the latter possibility was the occurrence of many mesopelagic species (mainly myctophids, hatchetfishes and deep-sea squids) floating dead at the surface during the strongest episodes of volcanic unrest (Escánez, pers. comm.). This is not surprising, because it is probable that the magnitude of the eruption did not leave scope for adaptation although deep-sea animals have a high tolerance threshold for varying oxygen [15] and temperature conditions [14]. Many mesopelagic organisms tolerate hypoxia by reducing their metabolism as the consequence of lower temperatures in the deep ocean [15], but in warmer waters, the oxygen consumption increases dramatically [46], [47]. Presumably, the oxygen demands of vertical migrants during feeding activity were therefore much higher than in existing reserves in shallow waters of El Hierro Island. Besides, it has recently been documented in myctophids (main group forming the MSL) that heat shock responses under warm conditions might be triggered by the oxidative stress that occurs in normoxic waters [48]. It thus seems likely that the natural plasticity of the migrant biota no longer worked under the atypical scenario of low oxygen and high temperature. This, along with adverse effects of ocean acidification [1], [49] and the presence of toxic chemical compounds, suggests that both vertical and horizontal evasion would be the only means to avoid death.

### The elevation of the Deep Scattering Layer

The presence of unusual acoustic scattering layers up to 200 m above the normal upper limit of the DSL might be interpreted both as an elevation of the DSL or as a lowering of the MSL. Since that phenomenon also occurred during daytime, when there was no migration, we are inclined to favor the former hypothesis although we do not reject the idea that during nighttime backscattering was also induced by arrested migrants. Those elevations were likely promoted by the light attenuation caused by the water turbidity at the surface. Chemical perturbations seemed to have a limited effect since the DSL was closer to the surface below turbid waters, even though no hypoxia was registered (St. 3 in Fig. 3). It remained, however, at normal depth coinciding with clean waters despite the oxygen and acoustic anomalies found at the surface (St. 5 in Fig. 4).

Light-induced vertical migrations of scattering layers are well documented although most of the information refers to time-based patterns [17], [50], [51] rather than to spatial variations. In discussions of temporal patterns, the difficulty of discerning to what extent behavior is controlled by an external stimulus (e.g., light irradiance) or by endogenous biological rhythms has been raised [52]. The pattern reported here is not a diel migration since it occurred all day and varied with geographic location rather than with time. Apparently, these vertical relocations were triggered by light alone (or indirect knock-on effects) but not by time. Daytime elevations of acoustic scattering layers have also previously been reported in relation to varying light, including variable cloudiness [18], [53] or harmonic migrations caused by shading from turbid surface layers that varied in thickness due to internal waves [54]. Corresponding to the present study, shading-induced migrations have also been documented in the spatial dimension. Sassa et al. (2010) observed patches with shallower distributions of the lanternfish *Benthosema pterotum* coinciding with turbid areas caused by suspended sediments [55], while Kaartvedt et al. (1996) reported shallow scattering layers below waters inside a front characterized by higher light extinction [56]. These unpredictable shading effects over the deep scattering biota show that although internal clocks might also operate, the external stimulus is a main trigger for the migrant behavior within the mesopelagic zone.

It is generally assumed that migrant planktivores increase their food intake as they move to upper layers but also that the mortality risk increases since the higher illumination in the upper layers exposes them to larger predators. Dawn and dusk provide intermediate levels of light intensity in upper waters, where the ratio of mortality risk to feeding rate reaches a minimum. These brief antipredation windows of time in upper waters [57] occur twice per day during the DVM. We suggest that fortuitous shadings in the water column, like those produced by the volcano eruption, might be exploited by migrants to stretch out the antipredation window and, therefore, to increase their feeding rate. Exploitation of additional feeding windows has also previously been suggested when high algal densities in upper layers diminish the light penetration in the water column [54], [56].

### The evolution of the pelagic biota along the volcanic unrest

Waters outside the main influence of the eruption, used here as control zones, were also somewhat affected since the satellite reflectances also registered some small turbidity pulses during the strongest eruptive episodes. This might explain the fact that phyto- and mesozooplankton indices were likewise low in the affected and the control zones, at least at the beginning of the monitoring. On the contrary, vertical migrant micronekton formed a stronger MSL in the control zone. A feasible explanation for the divergent responses observed between these communities outside the main plume could be that, in the case of phyto- and mesozooplankton, we were actually sampling organisms advected from the affected zone (the undermined stocks would reach the control zone in the same way as turbid waters did). In contrast, vertical migrant micronekton could avoid the water perturbations by remaining in deeper waters (see discussion about the weakening of the MSL). Once the adverse conditions were temporarily alleviated (between turbidity pulses), the migrants could again occupy the shallow waters while the epipelagic plankton community would be restoring its population.

According to the biological indices analyzed, it seems clear that plankton and nekton in shallow waters were negatively affected during the first and strongest episodes of the volcanic unrest. In situ chlorophyll *a* values were 0.04–0.05 mg m^−3^ while the values in the Canary Islands, during the same season (stratified water column and at the leeward side of the island), ranged from 0.06 to 0.34 [58]. Our values were even lower than those collected by Davenport et al. (2002) during the same season in oligotrophic oceanic waters northward of the archipelago (0.09 mg m^−3^) [24]. Epipelagic mesozooplankton densities were certainly low (2.8–4.4 mg DW m^−3^) but not so different from those reported by Arranz (2007) around the volcano area before the eruption and during the same season (4.4–13.1 mg DW m^−3^) [26]. The vertical migrant micronekton was also affected as suggested by the weak MSL in the zone influenced by the eruption compared to the control zone.

All biological parameters increased slightly in magnitude immediately after the eruption stopped. Whether these enhancements were caused by a potential fertilization due to the volcano eruption [7], or to natural inputs of nutrients due to winter mixing, is difficult to know unless we study changes in nutrients field over the course of the study across the whole region. Nevertheless, the cessation of the eruption coincided with the lowest temperatures in the water column (February), a period otherwise characterized by the so-called late winter bloom [59], [60]. This natural bloom is related to weakening of the thermocline as a result of surface cooling, allowing a small vertical flux of nutrients, and therefore, promoting biological production. Thus, the observed biological growth might be due to the late winter bloom as well as to fertilization by the volcano. After the eruption, *in situ* chlorophyll *a* in the affected area ranged from 0.17 to 0.35 mg m^−3^ (0.11–0.24 from remote sensing data) whereas Arístegui et al. (1997) obtained values between 0.16–0.77 during the late winter bloom, leeward of Gran Canaria Island [58]. The same author reported a maximum of 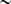
1.12 mg m^−3^ during a bloom in March in a time-series also performed in the Canary Islands [36]. In sum, we consider that the concentration of chlorophyll *a* during the post-eruptive phase was not above the normal range observed during this season in the Canary waters.

Concerning epipelagic mesozooplankton: in February, we obtained biomass values ranging from 4.1 to 6.8 mg DW m^−3^ whereas, in the same zone and before the volcano eruption, Arranz (2007) obtained higher values during a bloom in April-March (6.0–14.3 mg DW m^−3^) [26]. Our values were also lower than those compiled by Hernández-León (2007) for the bloom conditions in the Canary Islands (8.7–13.5) [61]. Thus, as in the case of chlorophyll *a*, the mesozooplankton biomass was higher after the volcano eruption, but within the normal range for this season. On the other hand, during the post-eruptive phase, all the biological parameters were somewhat higher in the volcano-affected area than in the control zone (except mesozooplankton in terms of biomass). We attribute this increment to an island-mass effect [62] rather than to volcano fertilization. This phenomenon produces richer nutrient waters as a result of the current flow perturbations occurring in the downstream wakes of the islands. Island-mass effects have previously been documented in the Canary Islands and with stronger gradients of biological production by Hernández-León (1988) [63] and Arístegui et al. (1997) [58], who observed biomass increments (up to three-fold higher) in both chlorophyll *a* and mesozooplankton as they approached to the wake of Gran Canaria Island. According to our values and baseline levels around the coastal Canary waters, we found no evidence of a volcano-induced bloom during the post-eruptive phase. We posit that surface chlorophyll *a*, epipelagic mesozooplankton and vertical migrant micronekton simply restored to normal levels in the area.

In summary, biological monitoring that ran in parallel with a submarine volcanic episode provided valuable observations of both adverse effects and adaptive responses by pelagic biota. The environmental stressors accompanying the eruption promoted the depletion of epipelagic stocks, changed the vertical distribution of the deep scattering biota and interrupted the diel vertical migration. In contrast, the post-eruptive phase indicated the restoration capacity of pelagic ecosystems after volcanic perturbations. We underline the importance of studying these phenomena as they provide a valuable understanding of how the marine ecosystem responds to environmental stressors.

## References

[1] Hall-Spencer JM, Rodolfo-Metalpa R, Martin S, Ransome E, Fine M, et al (2008) Volcanic carbon dioxide vents show ecosystem effects of ocean acidification. Nature 454: 1–4. 10.1038/nature07051 18536730

[2] Mantas VM, Pereira A, Morais PV (2011) Plumes of discolored water of volcanic origin and possible implications for algal communities. The case of the Home Reef eruption of 2006 (Tonga, Southwest Pacific Ocean). Remote Sensing of Environment 115: 1341–1352.

[3] Olgun N, Duggen S, Langmann B, Hort M, Waythomas C, et al (2013) Geochemical evidence of oceanic iron fertilization by the Kasatochi volcanic eruption in 2008 and the potential impacts on Pacific sockeye salmon. Marine Ecology Progress Series 488: 81–88.

[4] Hamme RC, Webley PW, Crawford WR, Whitney Fa, DeGrandpre MD, et al. (2010) Volcanic ash fuels anomalous plankton bloom in subarctic northeast Pacific. Geophysical Research Letters 37..

[5] Rivera J, Lastras G, Canals M, Acosta J, Arrese B, et al (2013) Construction of an oceanic island: Insights from the El Hierro (Canary Islands) 20112012 submarine volcanic eruption. Geology 41: 355–358.

[6] Fraile-Nuez E, González-Dávila M, Santana-Casiano JM, Arístegui J, Alonso-González IJ, et al. (2012) The submarine volcano eruption at the island of El Hierro: physical-chemical perturbation and biological response. Scientific Reports 2.. 10.1038/srep00486 PMC3390001 22768379

[7] Santana-Casiano JM, González-Dávila M, Fraile-Nuez E, de Armas D, González AG, et al. (2013) The natural ocean acidification and fertilization event caused by the submarine eruption of El Hierro. Scientific reports 3.. 10.1038/srep01140 PMC3555091 23355953

[8] Dietz RS (1948) Deep scattering layer in the Pacific and Antarctic Oceans. Journal of marine research 7: 430–442.

[9] Tucker GH (1951) Relation of fishes and other organisms to the scattering of underwater sound. Journal of marine research 10: 215–238.

[10] Robinson C, Steinberg DK, Anderson TR, Arístegui J, Carlson CA, et al (2010) Mesopelagic zone ecology and biogeochemistry–a synthesis. Deep Sea Research Part II: Topical Studies in Oceanography 57: 1504–1518.

[11] Barham EG (1966) Deep scattering layer migration and composition: Observations from a diving saucer. Science 151: 1399–1403. 17817303 10.1126/science.151.3716.1399

[12] Roe HSJ (1974) Observations on the diurnal vertical migrations of an oceanic animal community. Marine Biology 28: 99–113.

[13] Pearre S (2003) Eat and run? The hunger/satiation hypothesis in vertical migration: History, evidence and consequences. Biological Reviews of the Cambridge Philosophical Society 78: 1–79. 12620061 10.1017/s146479310200595x

[14] Watanabe H, Moku M, Kawaguchi K, Ishimaru K, Ohno A (1999) Diel vertical migration of myctophid fishes (Family Myctophidae) in the transitional waters of the western North Pacific. Fisheries Oceanography 8: 115–127.

[15] Ekau W, Auel H, Pörtner HO, Gilbert D (2010) Impacts of hypoxia on the structure and processes in pelagic communities (zooplankton, macro-invertebrates and fish). Biogeosciences 7: 1669–1699.

[16] Bianchi D, Galbraith ED, Carozza DA, Mislan KAS, Stock C (2013) Intensification of open-ocean oxygen depletion by vertically migrating animals. Nature Geoscience 6: 545–548.

[17] Roe HSJ (1983) Vertical distributions of euphausiids and fish in relation to light intensity in the Northeastern Atlantic. Marine Biology 77: 287–298.

[18] Staby A, Aksnes D (2011) Follow the light-diurnal and seasonal variations in vertical distribution of the mesopelagic fish Maurolicus muelleri. Marine Ecology Progress Series 422: 265–273.

[19] Angel MV, Pugh PR (2000) Quantification of diel vertical migration by micronektonic taxa in the northeast Atlantic. Hydrobiologia 440: 161–179.

[20] Hidaka K, Kawaguchi K, Murakami M, Takahashi M (2001) Downward transport of organic carbon by diel migratory micronekton in the western equatorial pacific: Its quantitative and qualitative importance. Deep-Sea Research Part I: Oceanographic Research Papers 48: 1923–1939.

[21] Davison P, Checkley D, Koslow J, Barlow J (2013) Carbon export mediated by mesopelagic fishes in the northeast Pacific Ocean. Progress in Oceanography 116: 14–30.

[22] Schukat A, Bode M, Auel H, Carballo R, Martin B, et al (2013) Pelagic decapods in the northern Benguela upwelling system: Distribution, ecophysiology and contribution to active carbon flux. Deep Sea Research Part I: Oceanographic Research Papers 75: 146–156.

[23] Irigoien X, Klevjer TA, Rø stad A, Martinez U, Boyra G, et al. (2014) Large mesopelagic fishes biomass and trophic efficiency in the open ocean. Nat Commun 5.. 10.1038/ncomms4271 PMC3926006 24509953

[24] Davenport R, Neuer S, Helmke P, Perez-Marrero J, Llinas O (2002) Primary productivity in the northern Canary Islands region as inferred from SeaWiFS imagery. Deep Sea Research Part II: Topical Studies in Oceanography 49: 3481–3496.

[25] Barton ED, Arístegui J, Tett P, Cantón M, García-Braun J, et al (1998) The transition zone of the Canary Current upwelling region. Progress in Oceanography 41: 455–504.

[26] Arranz P (2007) Composición y distribución del ictioplancton de la reserva marina de El Hierro, Islas Canarias. MS Thesis, Universidad de Las Palmas de Gran Canaria.

[27] Tuya F, García-Díez C, Espino F, Haroun RJ (2006) Assessment of the effectiveness of two marine reserves in the Canary Islands (eastern Atlantic). Ciencias Marinas 32..

[28] Arranz P, de Soto NA, Madsen PT, Brito A, Bordes F, et al (2011) Following a foraging fish-finder: diel habitat use of Blainville's beaked whales revealed by echolocation. PloS one 6: e28353. 22163295 10.1371/journal.pone.0028353 PMC3233560

[29] UNESCO (1994) Protocols for the Joint Global Ocean Flux Study (JGOFS) Core Measurements. Technical report, Intergovernmental Oceanographic Commission, UNESCO, Paris.

[30] Nechad B, Ruddick K, Park Y (2010) Calibration and validation of a generic multisensor algorithm for mapping of total suspended matter in turbid waters. Remote Sensing of Environment 114: 854–866.

[31] Doxaran D, Froidefond JM, Castaing P, Babin M (2009) Dynamics of the turbidity maximum zone in a macrotidal estuary (the Gironde, France): Observations from field and MODIS satellite data. Estuarine, Coastal and Shelf Science 81: 321–332.

[32] Claustre H, Morel A, Hooker SB, Babin M, Antoine D, et al. (2002) Is desert dust making oligotrophic waters greener? Geophysical Research Letters 29..

[33] Lin IL, Hu C, Li YH, Ho TY, Fischer TP, et al (2011) Fertilization potential of volcanic dust in the low-nutrient low-chlorophyll western North Pacific subtropical gyre: Satellite evidence and laboratory study. Global Biogeochemical Cycles 25: 1–12.

[34] MacLennan DN, Simmonds EJ (1992) Fisheries Acoustics: Theory and Practice. London: Chapman & Hall.

[35] Foote K, Knudsen H, Vestnes G, MacLennan D, Simmonds E (1987) Calibration of acoustic instruments for fish density estimation: a practical guide. ICES Cooperative Research Report 144..

[36] Grosjean P, Denis K (2007) Zoo/PhytoImage version 1.2-0. User's Manual.

[37] Lehette P, Hernández-León S (2009) Zooplankton biomass estimation from digitized images: a comparison between subtropical and Antarctic organisms. Limnology and Oceanography: Methods 7: 304–308.

[38] Troupin C, Barth A, Sirjacobs D, Ouberdous M, Brankart JM, et al (2012) Generation of analysis and consistent error fields using the Data Interpolating Variational Analysis (DIVA). Ocean Modelling 52–53: 90–101.

[39] Stanton TK, Chu D (2000) Review and recommendations for the modelling of acoustic scattering by fluid-like elongated zooplankton: euphausiids and copepods. ICES Journal of Marine Science 57: 793–807.

[40] Simmonds E, MacLennan D (2005) Fisheries Acoustics: Theory and Practice. London: Blackwell Science Ltd.

[41] Love RH, Fisher Ra, Wilson Ma, Nero RW (2004) Unusual swimbladder behavior of fish in the Cariaco Trench. Deep Sea Research Part I: Oceanographic Research Papers 51: 1–16.

[42] Wienerroither R, Uiblein F, Bordes F, Moreno T (2009) Composition, distribution, and diversity of pelagic fishes around the Canary Islands, Eastern Central Atlantic. Marine Biology Research 5: 328–344.

[43] Bordes F, Wienerroither R, Uiblein F, Moreno T, Bordes I, et al.. (2009) Catálogo de especies meso y batipelágicas. Peces, moluscos y crustáceos. Colectadas con arrastre en las Islas Canarias durante las campañas realizadas a bordo del B/E “La Bocaina”. Instituto Canario de Ciencias Marinas.

[44] Korneliussen R (2000) Measurement and removal of echo integration noise. ICES Journal of Marine Science 57: 1204–1217.

[45] Korneliussen RJ, Heggelund Y, Eliassen IK, Johansen GO (2009) Acoustic species identification of schooling fish. ICES Journal of Marine Science 66: 1111–1118.

[46] Donnelly J, Torres JJ (1988) Oxygen consumption of midwater fishes and crustaceans from the eastern Gulf of Mexico. Marine Biology 97: 483–494.

[47] Torres JJ, Belman BW, Childress JJ (1979) Oxygen consumption rates of midwater fishes as a function of depth of occureence. Deep Sea Research Part A Oceanographic Research Papers 26: 185–197.

[48] Lopes AR, Trübenbach K, Teixeira T, Lopes VM, Pires V, et al.. (2013) Oxidative stress in deep scattering layers: heat shock response and antioxidant enzymes activities of myctophid fishes thriving in oxygen minimum zones. Deep Sea Research Part I: Oceanographic Research Papers.

[49] Fabry VJ, Seibel BA, Feely RA, Orr JC (2008) Impacts of ocean acidification on marine fauna and ecosystem processes. ICES Journal of Marine Science: Journal du Conseil 65: 414–432.

[50] Boden BP, Kampa EM (1967) The influence of natural light on the vertical migrations of an animal community in the sea. Symp Zool Soc Lond 19..

[51] Clarke GL, Backus RH (1953) Measurements of light penetration in relation 494 to vertical migration and records of luminescence of deep-sea animals. Deep Sea Research 4: 1–14.

[52] Neilson JD, Perry RI (1990) Diel Vertical Migrations of Marine Fishes: an Obligate or Facultative Process? Advances in Marine Biology 26: 115–168.

[53] Baliño BM, Aksnes D (1993) Winter distribution and migration of the sound scattering layers, zooplankton and micronekton in Masfjoerden, western Norway. Marine Ecology Progress Series 102: 35–50.

[54] Kaartvedt S, Klevjer TA, Aksnes DL (2012) Internal wave-mediated shading causes frequent vertical migrations in fishes. Marine Ecology Progress Series 452: 1–10.

[55] Sassa C, Tsukamoto Y, Yamamoto K, Tokimura M (2010) Spatio-temporal distribution and biomass of Benthosema pterotum (Pisces: Myctophidae) in the shelf region of the East China Sea. Marine Ecology Progress Series 407: 227–241.

[56] Kaartvedt S, Melle W, Knutsen T, Skjoldal HR (1996) Vertical distribution of fish and krill beneath water of varying optical properties. Marine Ecology Progress Series 136: 51–58.

[57] Clark CW, Levy DA (1988) Diel vertical migrations by juvenile sockeye salmon and the antipredation window. The American Naturalist 131: 271–290.

[58] Arístegui J, Tett P, Hernández-Guerra A, Basterretxea G, Montero MF, et al (1997) The influence of island-generated eddies on chlorophyll distribution: a study of mesoscale variation around Gran Canaria. Deep Sea Research Part I 44: 71–96.

[59] Arístegui J, Hernández-León S, Montero MF, Gómez M (2001) The seasonal planktonic cycle in coastal waters of the Canary Islands. Scientia Marina 65: 51–58.

[60] Hernández-León S, Almeida C, Bécognée P, Yebra L, Arístegui J (2004) Zooplankton biomass and indices of grazing and metabolism during a late winter bloom in subtropical waters. Marine Biology 145: 1191–1200.

[61] Hernández-León S, Gómez M, Arístegui J (2007) Mesozooplankton in the Canary Current System: The coastal ocean transition zone. Progress in Oceanography 74: 397–421.

[62] Doty MS, Oguri M (1956) The Island Mass Effect. Journal du Conseil 22: 33–37.

[63] Hernández-León S (1988) Gradient of mesozooplankton biomass and ETS activity in the windshear area as evidence of an island mass effect in the Canary Island waters. Journal of Plankton Research 10: 1141–1154.

